# A Survey of Exposure Level and Lifestyle Factors for Perfluorooctanoate and Perfluorooctane Sulfonate in Human Plasma from Selected Residents in Korea

**DOI:** 10.3390/ijerph110707231

**Published:** 2014-07-16

**Authors:** Jinhee Eom, Jaeyeon Choi, Jiye Kim, Yunje Kim

**Affiliations:** 1 Environmental Technology Research Center, Korea Institute of Science and Technology, P.O. Box 131, Cheongryang, Seoul ASI|KR|KS013|SEOUL, Korea; E-Mails: 114008@kist.re.kr (J.E.); 114033@kist.re.kr (J.C.); 114001@kist.re.kr (J.K.); 2 Department of Chemistry, Korea University, Anam-ro, Seongbuk-gu, Seoul 136701, Korea

**Keywords:** perfluorooctanoate (PFOA), perfluorooctane sulfonate (PFOS), liquid chromatography time-of-flight mass spectrometry (LC/TOF-MS)

## Abstract

Following few decades of commercial use, perfluorooctanoate (PFOA) and perfluorooctane sulfonate (PFOS) have been found in human blood and serum. We determined the amounts of PFOA and PFOS in human plasma (*n* = 183) and the effects of multiple uses of food-contact materials and smoking habits and alcohol consumption using liquid chromatography time-of-flight mass spectrometry (LC/TOF-MS). For the paper cups, the PFOA level in the plasma of the heavy user group was 1.37 times higher than that of the light user group. However, no association between the effects of multiple uses of food-contact materials and the plasma levels of PFOA and PFOS was found, except for paper cups. Active smokers had lower plasma levels of PFOA and PFOS than non-smokers. We show that multiple uses of food-contact materials do not appear to be a significant source of PFOA and PFOS.

## 1. Introduction

Perfluorooctanoate (PFOA) and perfluorooctane sulfonate (PFOS) are classified as perfluorinated compounds (PFCs) and were manufactured for their unique chemical stability and surface-tension lowering properties [1,2]. These synthetic compounds have been used for the past few decades and have been discovered to be globally distributed and persistent environmental contaminants [3]. In addition, detectable concentrations of PFOS and PFOA have been found globally in many wildlife species, as well as open ocean waters [4]. Many studies have reported the occurrence of PFOA and PFOS in the blood of persons in the general population [5,6]. In addition to this, evidence that PFOA and PFOS can cause endocrine disruption and developmental toxicity has been reported [7]. Therefore, there is a need to identify the sources of exposure of PFOA and PFOS in the general population. Due to its oil and water repellent properties, the ammonium salt of PFOA has been used as an essential additive in the manufacture of polytetrafluoroethylene (PTFE) for non-stick cookware, anti-static products, and products in the electronic industry [8]. In particular, PTFE is known to have a high degradation point (327 °C) and to be extremely chemically resistant to a large variety of chemicals [9]. In addition, fabrics, carpets, paper coatings, surfactants, and cosmetics have been considered as potential exposure pathways for PFCs [10]. Not only a major commercial product, PFOS has also been used in some products, including fire-fighting foams [11]. However, the temperatures reached during the use of commercial products are less than the thermal degradation point. Residual PFOA has been detected in the surface coating of nonstick cookware [12]. The release of such a residual emulsifier provides a potential source of human exposure to PFOA and PFOS. Additionally intake of red meat, animal fats, and snacks are important predictors of plasma levels of PFOS due to contamination from leaching of paper coatings [13]. This paper describes the determination of the amounts of PFOA and PFOS in 183 human plasma samples (Table 1). The association between plasma levels of PFOA and PFOS and different commercial products (mostly food-contact materials) as well as the migration characteristics of PFOA and PFOS from actual food-contact materials (paper cups, plastic wrap and zip-lock containers, disposable containers, airtight containers, PTFE-coated cookware, and antistatic materials) was studied. Additionally, potential relationships between smoking habits and alcohol consumption and levels of PFOA and PFOS in the comparison of regional areas were studied.

**Table 1:** The amounts of PFOA and PFOS in 183 human plasma (ng/mL).

Sample		Residents		Industrial	
		Metropolis	Rural	Industrial	Sum
Gender	Man	7	38	17	62
	Woman	18	100	3	121
Age	20–29	7	10	3	20
	30–39	8	40	0	48
	40–49	4	45	10	59
	50	6	43	7	56
Sum		25	138	20	183
Smoking	Smoker	4	24	8	36
(only man)	Nonsmoker	3	14	9	26
Drinking	Drinker	2	10	3	15
(only man)	Nondrinker	5	28	14	47
Sum		7	38	17	62

## 2. Experimental Section

### 2.1. Chemicals

PFOS was purchased from Fluka (Steinheim, Swizerland). PFOA and tridecafluoroheptanoic acid (PFHpA) as internal standard (ISTD) were purchased from Aldrich (St. Louis, MO, USA). Stock solutions of each compound were prepared with methanol at a concentration of 1000 mg/L and stored at 4 °C. HPLC grade methanol used for solid phase extraction (SPE) and as mobile phase was purchased from Burdick & Jackson (Muskegon, MI, USA). Acetonitrile (ACN) used for deproteinization was purchased from Merck (Darmstadt, Germany). Tetrabutylammonium hydrogensulfate (TBA) as ion pairing agent was purchased from Aldrich. Ammonium acetate added to the mobile phase was obtained from Yakuri Pure Chemical (Kyoto, Japan).

### 2.2. Volunteer Samples

The plasma samples (*n* = 183) were provided from a medical center of Yonsei University from volunteer donations. Ethical approval has been granted by the ethical committee of the Yonsei University Hospital. Either participants or guardians provided a written informed consent for study participation. Samples were stored in a glass tube at −20 °C until the laboratory analysis. Participants in this study were asked to fill out a questionnaire to help organize the samples and interpret the results. The questionnaire was used to obtain personal information (sex, age, weight, height, occupation, medical history and location) and lifestyle status (the use of paper cups, antistatic materials, plastic wrap, airtight containers, and PTFE-coated cookware; habits of drinking and smoking were also objects of the questionnaire.

### 2.3. Extraction and Derivatization Procedure

50 μL of PFHpA solution (10 μg/mL of PFHpA in methanol) as ISTD was spiked with 5 mL of plasma, and solution was adjusted to pH 10 by adding 100 mg of K_2_CO_3_. ACN (10 mL) was added to this plasma sample for protein removal, and the mixture was shaken and centrifuged at 400 g for 10 min. The upper fraction of the liquid was retrieved and 50% formic acid (3 mL) was added to prevent the cartridge from clogging up during the SPE process. This resulting solution was concentrated to 2 mL with nitrogen gas. A C_18_ cartridge was previously activated and conditioned with distilled water (DW, 5 mL), methanol, and tetrabutylammonium hydrogensulfate (TBA) prior to its use. The concentrated solution was applied to the C_18_ cartridge for solid-phase extraction. Then the cartridge was washed with DW (5 mL), and the analytes were eluted three times with methanol (2 mL) and evaporated to dryness under a stream of nitrogen. Finally, the effluent was dissolved in 50 μL of methanol.

### 2.4. Instrument and Equipment

A LC/TOF-MS system, which consisted of an Agilent HP 1100 series HPLC (Palo Alto, CA, USA) and LECO Unique TOF-MS (St. Joseph, MI, USA), were used to detect the target compounds. The column was a Shiseido UG 120V C_18_ column (2.0 mm I.D × 150 mm length, particle size 5 μm). An HS501D shaker from IKA (Staufen, Germany) was used to mix the sample and the organic solvent. A Varifuge-F system from Heraeus (Hanau, Germany) was used for centrifugation. A vortex mixer from Scientific Industry (Bohemia, NY, USA) was used for mixing, and a Turbovap^®^ LV evaporator from Zymark (Hopkinton, MA, USA) was used to dry the samples. Rapid Trace SPE used for solid phase extraction was purchased from Caliper (Hopkinton, MA, USA), and an AccBond II ODS-C18 cartridge (200 mg, 3 mL) was purchased from Agilent. The LC/TOF-MS was operated with electro-spray ionization (ESI) voltage −3.3 kV, and desolvation gas flow of 7000 mL/min with nitrogen gas; this system detected the negative ions. The mobile phase A consisted of 2 mM ammonium acetate in water, phase B was 2 mM ammonium acetate in methanol. The gradient time table is shown as followed:

Operating condition for HPLC/ESI/TOF/MS:

**HPLC Conditions:**

Column: Shiseido, UG120, C18 (2.0 mm i.d. × 150 mm length, particle size 5 μm)

Flow Rate: 0.3 mL/min

Mobile Phase Solvent A: 2 mM ammonium acetate in water; 

Solvent B: 2 mM ammonium acetate in methanol

Gradient time table: 

Time (min) A solvent (%) B solvent (%)050502505051585819915199205050305050

Run Time: 30 min

Injection Volume: 20 μL

Column Temp.: 40 °C

**ESI/TOF/MS Conditions:**

Ion Source Type: ESI (electrospray ionization)

Ionization Mode: negative ion mode

Electrospray Voltage: −3300 V

Desolvation Gas Flow: 7000 cc/min (N_2_) 

Desolvation Gas Temp.: 300 °C

Interface Temp.: 120 °C

## 3. Results and Discussion

### 3.1. Determination of Target Analytes and ISTD

**Figure 1:**
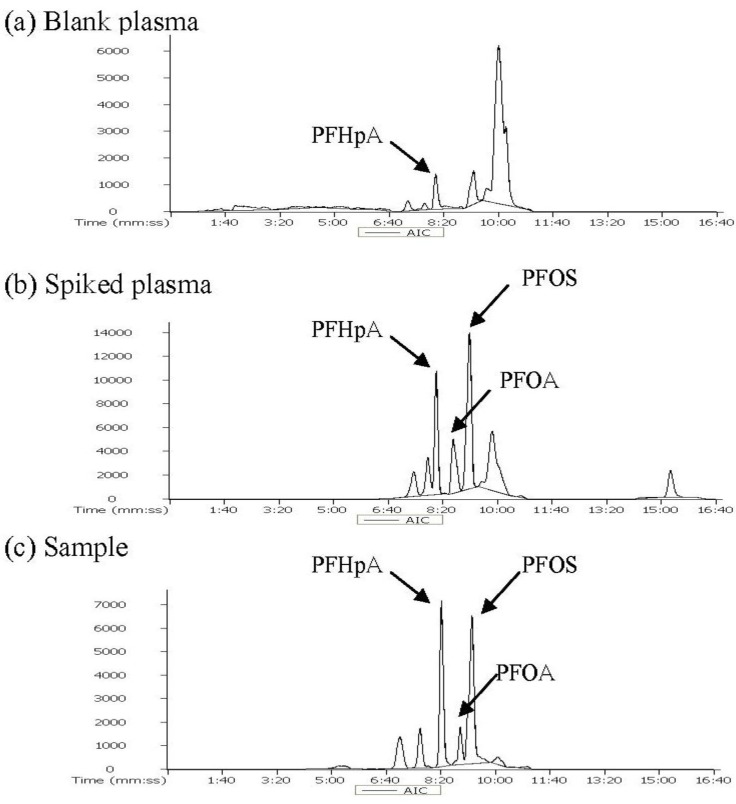
Adjusted ion chromatograms of PFOS and PFOA in (**a**) blank plasma; (**b**) spiked plasma and (**c**) sample by LC/ESI/TOF/MS.

**Figure 2:**
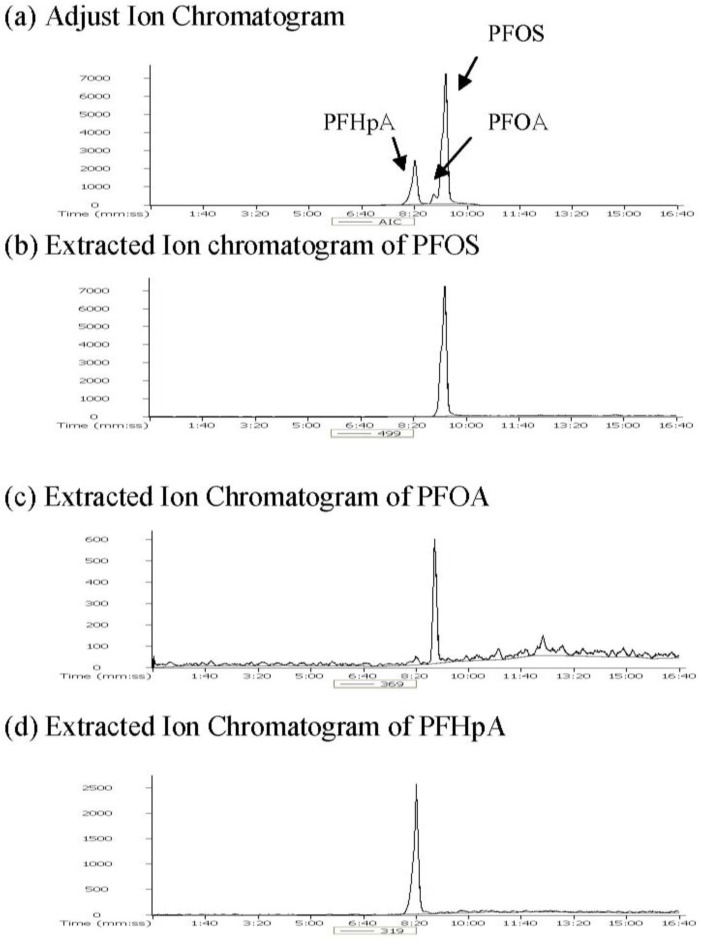
(**a**) Adjusted ion chromatogram and extracted chromatograms of (**b**) PFOS; (**c**) PFOA, (**d**) PFHpA (ISTD) from standard solution.

PFOA, PFOS, and PFHpA were separated and the results are shown in the adjusted total ion chromatograms (AIC) of the blank plasma, spiked plasma and samples seen in Figure 1. Extracted ion chromatogram of PFOA, PFOS and PFHpA as an internal standard are shown in Figure 2, and the separation of PFOA (8.56 min), PFOS (9.19 min) and PFHpA (8.21 min) was confirmed.

### 3.2. Validation of Analytical Method for PFOA and PFOS in Plasma

The experimental method used on the plasma samples was validated in terms of its recovery and reproducibility using LC/TOF-MS. The recovery of PFOA was 78.9%; that of PFOS was 97.2%. And, the reproducibility of PFOA and PFOS were 3.3% and 10.0%. The standard calibration curves for PFOA (*r*^2^ > 0.9877) and PFOS were (*r*^2^ > 0.9933) were linear from 0.5–10 ng/mL with a useful correlation coefficient. The limit of quantification (LOQ) of PFOS was 0.4 ng/mL and that of PFOA was 0.6 ng/mL.

### 3.3. Potential Relationships between the Plasma Levels of PFOA and PFOS and the Multiple Uses of Food-Contact Commercial Materials

To examine the factors contributing to the occurrence of PFOA and PFOS in participants, we searched for potential relationships between the levels of PFOA and PFOS and the multiple uses of chemical products. PFOA and PFOS have been widely used in coating paper, food-contact materials, and antistatic materials. The measured data in this study were grouped into a light group (light user: 1~4 times in a month), a medium group (1~4 times in a week) and a heavy group (heavy user: 1~3 times a day) according to the use of each type of product; these categories are shown in Table 2. Specifically, paper coated materials are typically heated to over 100 °C in about 1 min. These temperatures significantly increase the potential for migration of the packaging components to food. The PFOA level in the plasma of the heavy user group (1.69 ng/ mL) was 1.37 times higher than the level of people in the light user group (1.23 ng/mL); this was a, statistically significant difference (*p* = 0.0257). The multiple uses of paper cups are significantly correlated to exposure to PFOA level. PFOS level in the plasma of the heavy user group (3.89 ng/mL) was 1.22 times higher than that of people in the light user group (3.17 ng/mL), but this value did not represent a statistically significant difference (*p* = 0.6644). These values are in good agreement with those of earlier studies. Popcorn and fast food may be indirect dietary sources of chemicals due to contamination from leaching of paper coatings [13].

**Table 2:** The plasma levels of PFOA and PFOS and the multiple uses of food-contact commercial materials (ng/mL).

Material	Intensity	Mean	Range	*p*-value	Mean	Range	*p*-value
Paper cup	Light (*n* = 66)	1.23	N.D.~3.07	0.0257	3.81	1.14~11.80	0.6644
	Intermediate (*n* = 58)	1.7	N.D.~5.28		4.14	1.17~10.71	
	Heavy (*n* = 55)	1.69	N.D.~8.05		3.89	0.97~10.39	
Wrap and zip-lock bag	Light (n = 80)	1.5	N.D.~8.05	0.6921	3.99	0.97~11.80	0.1863
	Intermediate (*n* = 58)	1.6	N.D.~5.28		4.14	1.26~8.64	
	Heavy (*n* = 22)	1.36	N.D.~2.56		3.17	1.23~6.32	
Disposable container	Light (*n* = 134)	1.44	N.D.~5.28	0.0412	4.03	0.97~11.80	0.3765
	Intermediate (*n* = 17)	2.08	N.D.~3.54		3.44	1.23~6.05	
	Heavy (*n* = 9)	1.3	N.D.~8.05		3.17	1.37~10.39	
Air tight container	Light (*n* = 36)	1.86	N.D.~8.05	0.0022	4.97	1.14~11.80	<0.0001
	Intermediate (*n* = 35)	1.69	N.D.~5.28		3.23	1.25~7.21	
	Heavy (n = 90)	1.23	N.D.~3.38		3.55	0.97~8.46	
Antistatic products	Light (*n* = 62)	1.79	N.D.~4.76	0.0003	4.21	0.97~9.31	0.0802
	Intermediate (*n* = 54)	1.11	N.D.~2.91		3.71	1.14~11.80	
	Heavy (*n* = 22)	1.18	N.D.~2.93		3.18	1.23~8.30	
PTFE coated cookware	Light (*n* = 35)	1.77	N.D.~3.81	0.0005	4.59	0.97~9.31	0.0143
	Intermediate (*n* = 80)	1.35	N.D.~3.54		3.51	1.14~11.80	
	Heavy (*n* = 21)	1.13	N.D.~2.56		2.99	1.23~6.24	

N.D.: not detected.

However, plastic wrap and zip-lock bags were not statistically correlated. The levels of PFOA (1.5 ng/mL) and PFOS (3.99 ng/mL) in plasma of the light user group were 1.1 (*p* = 0.6921) and 1.26 (*p* = 0.1863) times higher than those of heavy user group. Plastic wrap and zip-lock bags are used for food packaging and microwave cooking. In microwave uses, plastic wrap and zip-lock bags are heated to over 200 °C in about 1–2 min. Begley conducted experiments at 100 °C with 15 min contact time and found that the chemicals in such paper products can migrate into emulsions, such as butter, margarine, and chocolate spread [14]. As the consumption rates of butter, margarine, and chocolate are low in Korea, plastic wrap and zip-lock bags are not important routes of human exposure.

For an airtight container, the levels of PFOA (1.86 ng/mL) and PFOS (4.97 ng/mL) in plasma of light user group were 1.51 (*p* = 0.0022) and 1.4 (*p* < 0.0001) times higher than those of heavy user group. For disposable containers, the highest level of PFOA (*p* = 0.0412) and PFOS (*p* = 0.3765) was in the plasma of the medium user group (2.08 ng/mL) and the plasma of the light user group (1.3 ng/mL). Air tight containers and disposable containers are used at low temperature for food-storage in freezers. This suggests that the concentrations of PFOA and PFOS are not significantly affected by food packaging materials under the conditions required to store the foods.

For PTFE-coated cookware, levels of PFOA (1.77 ng/mL) and PFOS (4.21 ng/mL) in the plasma of the light user group were slightly higher than those of heavy user group. One study has reported that PFOA remains as a residual on the surface and may be off-gassed when heated at normal cooking temperatures, but not in this case. This result provides evidence that residual PFOA on the pan is not completely vaporized during the nonstick coating fabrication process. In addition to this, the mass of off-gassed PFOA released was found to decrease following multiple uses [15].

For the antistatic materials, the levels of PFOA (1.79 ng/mL) and PFOS (4.21 ng/mL) in the plasma of the light user group were 1.52 and 1.32 times higher than those of the heavy user group. This suggests that the use of antistatic may not explain the lower level of PFOA and PFOS.

Dietary intake, migration from packaged foods and non-stick cookware, and dust are assumed to be major routes of exposure for the general population. [7,14,16] However, a finding of actual PFOA migration from PTFE-coated cookware is not likely. Data in this study suggest that fluoropolymer food contact materials do not appear to be a significant source of PFOA and PFOS.

### 3.4. Potential Relationships between Smoking Habits and Drinking and Levels of PFOA and PFOS

We examined smoking status (current smoker, never smoker) and alcohol intake. We found that never smokers had higher levels of PFOA and PFOS than did active smokers. The each level is shown in Table 3. The level of PFOA (2.26 ng/mL) in the plasma of never smokers were 1.04 times higher than that in the plasma of current smokers. The level of PFOS (4.63 ng/mL) in the plasma of smokers was 1.2 times higher than that in the plasma of never smokers; however, these results were insignificant. We found no significant association between smoking intensity among smokers and PFOA and PFOS plasma levels. These results suggest that smoking status may not explain the lower level of PFOA and PFOS. A similar study was performed in Denmark. Active smokers had lower plasma levels of PFOA and PFOS than did people who had never smoked [14]. In addition to this, a Japanese study suggests that smoking status may not influence PFOA or PFOS levels [17]. These results could reflect differences between current smokers and never smokers in other routes associated with sources of PFOA and PFOS exposure.

A slightly inverse association with PFOS level was found for alcohol intake. The levels of PFOA (2.20 ng/mL) and PFOS (4.83 ng/mL) in the plasma of never drinkers were 1.02 and 1.05 times higher than those in the plasma of drinkers. The diuretic effect of alcohol and the resulting loss of fluid from the body could be a possible explanation. A study showed a slightly inverse association between alcohol intake and PFOA and PFOS plasma levels in Denmark [17]. Further studies are required to define the sources in other routes associated with source of PFOA and PFOS exposure.

**Table 3:** Levels of PFOA and PFOS according to smoking habits and alcohol consumption (ng/mL).

Category	Intensity	PFOA			PFOS		
		Mean	Range	*p*-value	Mean	Range	*p*-value
Drinking	Drinker (*n* = 43)	2.16	N.D.~8.05	0.742	4.61	N.D.~10.71	0.321
	Never drinker (*n* = 17)	2.20	N.D.~5.28		4.83	N.D.~8.71	
Smoking	Smoker (*n* = 45)	2.18	N.D.~8.05	0.158	4.63	N.D.~10.39	0.157
	Never smoker (*n* = 17)	2.26	N.D.~4.76		3.85	N.D~8.16	

N.D.: not detected.

### 3.5. Potential Relationships between Regional Areas and levels of PFOA and PFOS

The order of PFOA level is industrial area > rural area > metropolis and PFOS is industrial area > rural area > metropolis. The PFOS level was approximately three times higher than PFOA level in all regional groups. We suggest that reasons for the lowest level in metropolis are high user awareness about the risks of environmental materials or low exposure to PFOA and PFOS due to the lowest mean age. The reason for the highest level in industrial area is there was more exposure to PFOA and PFOS than other regions. In the case of industrial area, the exposure measures for these compounds matched common expectation (Table 4).

**Table 4:** Levels of PFOA and PFOS in regional areas (ng/mL).

Area	PFOA		PFOS	
	Mean	Range	Mean	Range
Industrial worked	2.19	N.D~3.87	6.57	4.98~10.71
Rural residents	1.55	N.D~8.05	3.82	1.14~11.80
Metropolitan residents	0.79	N.D~2.28	2.47	0.97~8.71

N.D.: not detected.

## 4. Conclusions

Our results showed that multiple use of paper cups appears to be a potential pathway to increased PFOA plasma levels but not PFOS plasma levels. Plastic wrap and zip-lock bags were not statistically correlated with both compounds. No association with PFOA and PFOS level was found for the use of airtight containers and disposable containers. This suggests that the concentrations of PFOA and PFOS are not significantly affected by food packaging materials under the conditions required to store the foods. In this study, the results suggest that fluoropolymer food-contact materials and antistatic products do not appear to be significant sources of PFOA and PFOS. Active smokers had lower plasma levels of PFOA and PFOS than never smokers. A slight inverse association with PFOS level was found for alcohol consumption. In the comparison of regional exposures, the level of PFOS and PFOA in metropolitan plasma was lower than that of other regions and that of industrial areas was higher than in other regions.
